# Transdiagnostic cognitive-behavioral group therapy for anxiety disorders: Therapists’ perception of group management in community-based care

**DOI:** 10.1192/j.eurpsy.2022.1919

**Published:** 2022-09-01

**Authors:** C. Durand, M. Provencher, P. Norton, P. Roberge

**Affiliations:** 1 Université de Sherbrooke, Médecine De Famille Et Médecine D’urgence, Sherbrooke, Canada; 2 Université Laval, École De Psychologie, Québec, Canada; 3 Cairnmillar Institute, Psychology, Hawthorn East, Australia

**Keywords:** Qualitative, Therapist, CBT, Anxiety disorders

## Abstract

**Introduction:**

Cognitive-behavioral therapy (CBT) is recognized as an effective treatment for anxiety disorders. Transdiagnostic group CBT (tCBT) targets cognitive and behavioural intervention strategies common to anxiety disorders. tCBT allows the treatment of a larger number of patients simultaneously and therapists only need to master a single intervention protocol. However, tCBT may present several challenges for therapists, particularly regarding group management.

**Objectives:**

To explore therapists’ perceptions and experience of group management during tCBT for mixed anxiety disorders.

**Methods:**

A qualitative study embedded in a randomized controlled trial of group tCBT (Roberge & Provencher; CIHR, 2015-2021). Semi-structured interviews were conducted with 18 of the 21 therapists to document their perceptions and to identify improvements for tCBT delivery. The data were analyzed using a deductive approach and based on the interactive cyclical process of data reduction, display and conclusion drawing.

**Results:**

Therapists raised the challenge of the heterogeneous characteristics of participants’ anxious profile, since they had to be creative to provide exercises that were suitable for a whole group. Exposure exercises, a key component of tCBT, were particularly affected by the composition of the groups. Previous group animation experience and the ability to establish a therapeutic alliance from a group perspective were important facilitators. Co-therapy also facilitated the intervention, since it allowed the therapists to be more vigilant to group dynamics and favored the organization of tCBT.

**Conclusions:**

This study highlights the importance of exploring therapists’ perceptions and experience about group management in order to identify facilitators and barriers of group tCBT in community-based care.

**Disclosure:**

No significant relationships.

